# Territorial Voluntary Aid Detachments

**Published:** 1909-11-06

**Authors:** 


### The Royal Army Medical Corps Section.

### TERRITORIAL VOLUNTARY AID DETACHMENTS.

In The Hospital of September 25 an account was given of the Territorial Clearing Hospitals, whose work is to take over the wounded from the field ambulances and minister to their needs until they can be transferred to the general hospitals and convalescent homes at the base. To accomplish this task satisfactorily implies duties of a varied and important character, for it involves further transport work and possibly the care of the sick and wounded for some time, as it may be necessary to provide for them rest and refreshment-stations, or even over-night accommodation ("stationary hospitals") along the lines of communication. The provision of such assistance can only be properly furnished by a trained *personnel*, and as this does not exist at present, steps are being taken to establish it. According to a War Office Memorandum, issued on August 16, 1909, to secretaries of Territorial County Associations in England and Wales, the form this assistance is to take is that of *Voluntary Aid Detachments*, and each Territorial County Association has been charged with the responsibility of their formation and organisation.

### Voluntary Aid Detachments.

These Voluntary Aid Detachments are to be of two classes, and are to be made up of men and women respectively. A men's detachment will number 62, of whom 14 will be officers—namely one commandant, one assistant-commandant, two medical officers, two pharmacists, two assistant-pharmacists, and four under-officers. In a women's detachment there will be 26 members, of whom six will be officers—namely one commandant, one assistant-commandant, one quartermaster, one assistant quartermaster, and two lady superintendents. One feature common to both these detachments is that they are so arranged that they can be divided into equal halves, each of which can be provided with proper supervision—thanks to the duplication of officers. In the case of a women's detachment, no restriction is laid down about the officers, who may be either women or men. Anyone over 17 years of age can join a Voluntary Aid Detachment, but it has been laid down as a qualification for membership in England and Wales that everyone enrolling must possess the First Aid Certificate and Home Nursing Certificate of the St. John's Ambulance Association. With the view of giving increased facilities for obtaining these certificates, the Ambulance Department of the Order of St. John of Jerusalem has made special arrangements for teaching these subjects on a wide scale. It has formed a Territorial Branch of the St. John's Ambulance Association, which will establish in each county a section whose duty it will be to form separate "detached classes" of men and women for instruction in first aid and home nursing. To the members of these classes the necessary certificates of qualification will be given after examination, thus making them eligible for enrolment in the Voluntary Aid Detachments. It is not anticipated that this preliminary qualification will interfere with the immediate formation of the detachments, as all registered medical men and medical women, all fully trained and certificated hospital nurses, and all trained pharmacists may join at once, while, in addition, there must be scattered throughout the country large numbers of the holders of the St. John's First Aid and Nursing Certificates who will also be available, and who will be only too ready to come forward and help on this necessary and patriotic movement.

### Details of Training.

As soon as a detachment has been formed on the above lines, with all its members efficient in first aid and home nursing, it will then proceed to acquire a knowledge of its special duties. These are connected with the removal of the sick and wounded from the field ambulances to the general hospitals, and are of a varied nature, comprising transport duties and the provision of temporary hospitals, and of rest stations and evacuating stations. The distinguishing feature of all this work is that it requires prompt action and the ability on the part of those engaging in it to improvise what is required out of the local resources at hand. Especially is this power of improvisation needed in the matter of transport and the provision of shelter for the sick and wounded detained on the lines of evacuation. Consequently, the training will include instruction as to how to prepare country carts and other vehicles for the removal of patients lying down, how to fit up empty vans and rolling stock for their conveyance by rail, and how to provide stretchers and other means for severe cases that require carrying by hand. Suggestions, too, will be given as to how country houses, farms, public buildings, or even whole villages may be converted into temporary hospitals should the necessity arise, and how rest stations may be formed for the refreshment and accommodation of the sick and wounded while detained *en route* to the general hospitals.

This further training of a detachment in its special duties will not rest with the St. John's Ambulance Association. It is to be undertaken by the British Red Cross Society through its county committees, to whom a special appeal has been made in a recently issued memorandum on the subject. This memorandum impresses on every county the necessity for forming these detachments, and suggests that courses of lectures should be given on (1) the improvisation of apparatus for the transport of sick and wounded, (2) first aid nursing, and (3) practical demonstrations in the several varieties of work to be done. Of these duties the transport of the wounded would naturally fall to the men's detachments, and the preparation of food and any nursing duties to those of the women. From this it follows that the men should be trained chiefly as stretcher bearers; but it has very properly been suggested that they should also, to some extent, have an acquaintance with male nursing, and that some of them should be instructed in clerical duties and in sanitary work, especially the disinfection of buildings, and field sanitation. In the same way, although the formation of rest stations and the preparation and serving of meals to the wounded will devolve mainly on the women's detachments, it is very essential that they should be taught field cooking, the preparation of invalid diets, the fitting-up for patients of small wards in suitable buildings, and be made competent to do duty in ambulance trains, if called upon.

For the raising of these Voluntary Aid Detachments a very definite machinery has been laid down by the War Office Memorandum. It requires that the local branch of the Red Cross Society within each county will appoint a County Director of these detachments, who will be controlled by and be responsible to the local branch for the efficiency of the detachments. It is he who will say how many detachments are to be raised in the county and where they are to be located, a very necessary provision, as no limit has been fixed to the number of such detachments in a county. Each detachment as it is formed will be registered by the Council of the British Red Cross Society, and be given a consecutive number by the War Office. As at present arranged, members of these detachments will not be required to wear any specific uniform, but to each individual will be given an identity certificate, and he will be entitled to wear a brassard or red cross badge in time of war, so as to become "recognised" under the Geneva Convention.

The steps taken to ensure the efficiency of these detachments seem adequate. Primarily, the county director is responsible in this matter to the local branch of the British Red Cross Society within the county, and the latter in its turn is responsible to the Territorial County Association. In addition, the British Red Cross Society has decided to appoint an organiser of experience, whose appointment shall be approved by the Secretary of State for War. It is intended that this organising secretary shall be a retired Royal Army Medical Corps Officer of high rank, and that he shall visit the various county branches, assist and advise in the organisation of the detachments, and secure for the movement generally that uniformity of training and arrangement which is essential in any body that is intended to provide such supplementary aid to the Territorial Medical Service as will enable it to meet all the demands made on it in time of invasion. At present the War Office Memorandum applies only to England and Wales, but negotiations are proceeding with the Scottish Branch of the Red Cross Society as to the establishment of similar Voluntary Aid Detachments in Scotland, and we learn that the arrangements are well advanced for carrying out their formation; permission is, however, being asked to group the counties into districts, so as to bring them more under control and facilitate working when mobilised for war.

We congratulate the British Red Cross Society in having definitely come forward and undertaken the formation of these Voluntary Aid Detachments. It is work like this that will make its existence known throughout the United Kingdom, and show it in a favourable light. We have always felt that the system of latent Red Cross Committees was a distinct disadvantage, and that there was so much necessary work to be done in peace time, that a more active policy was called for. On these grounds this new departure is in every way to be commended, and we are satisfied that the appeal now made to the country on behalf of these detachments will meet with a hearty response.

